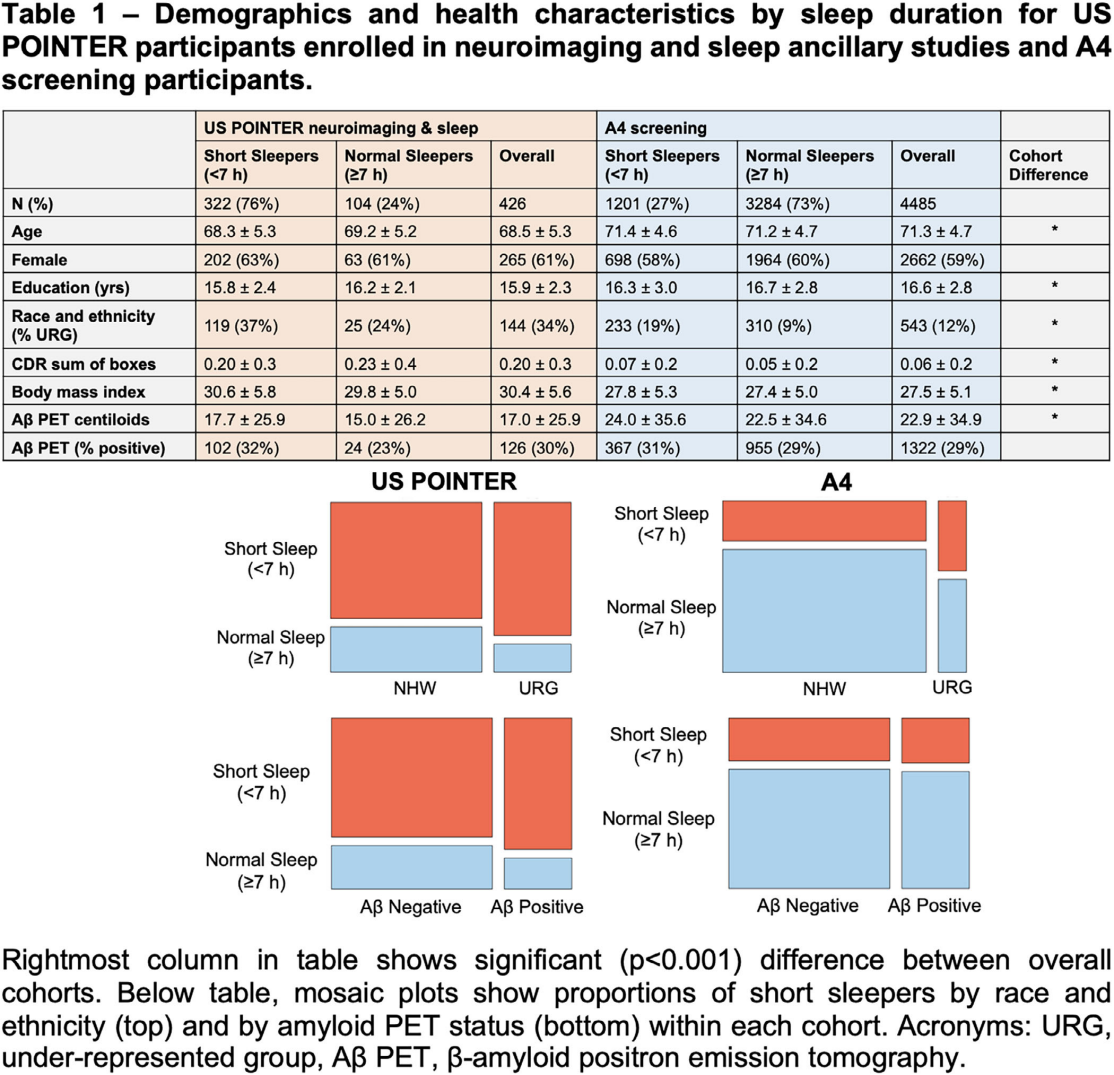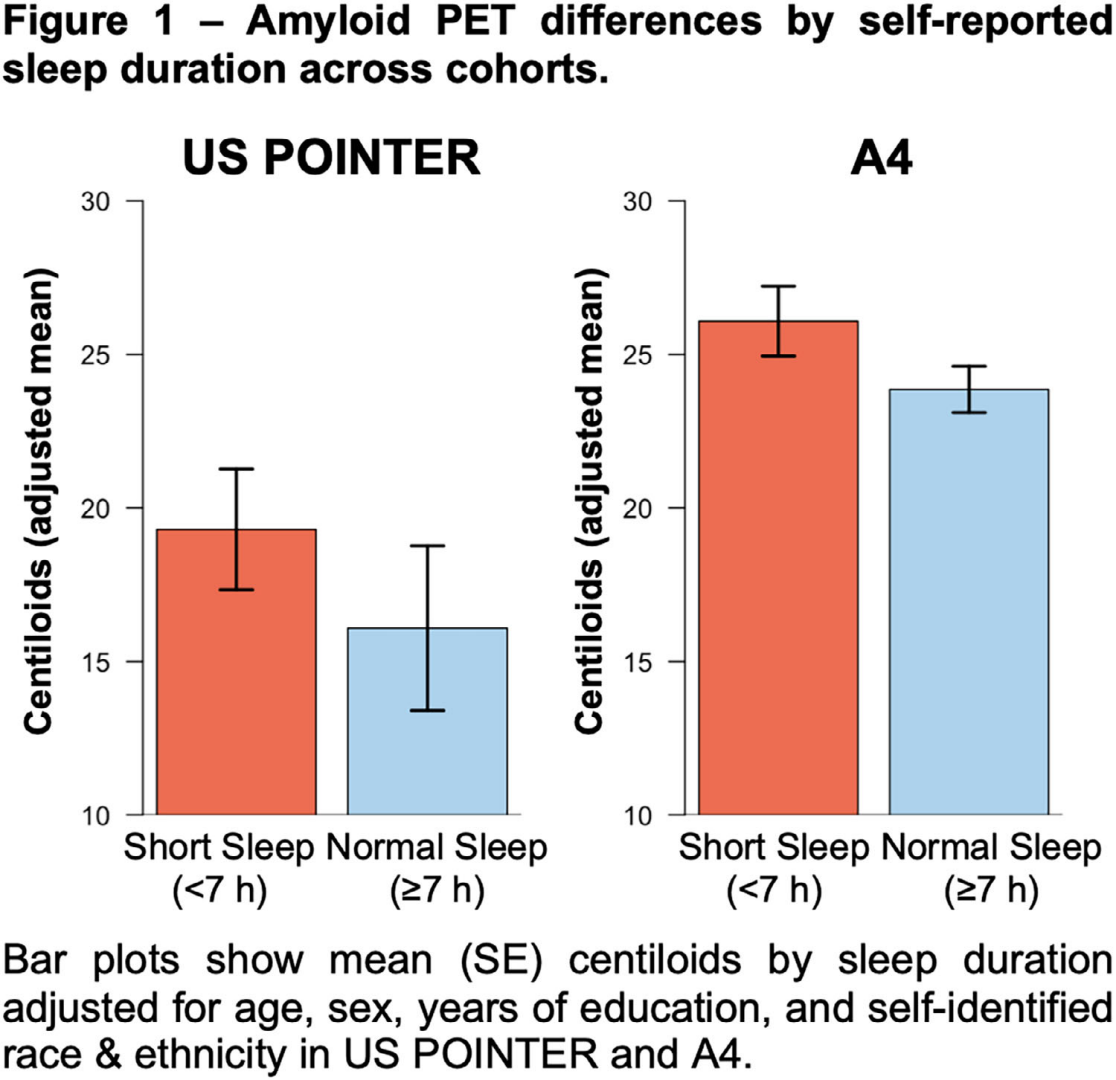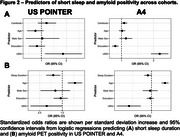# Heterogeneity in associations between short sleep duration and amyloid PET: Comparing US POINTER and A4

**DOI:** 10.1002/alz70856_100137

**Published:** 2025-12-25

**Authors:** Joseph R. Winer, Jacinda Taggett, Margaret Scales, Marjorie Howard, Katie L Stone, Stephanie Harrison, Sam N. Lockhart, Kathleen M. Hayden, Laura D Baker, Elizabeth C. Mormino, Susan M. Landau, Theresa M. Harrison, US POINTER Study Group

**Affiliations:** ^1^ Stanford University School of Medicine, Stanford, CA, USA; ^2^ University of California, Berkeley, Berkeley, CA, USA; ^3^ Wake Forest University School of Medicine, Winston‐Salem, NC, USA; ^4^ University of California, San Francisco, San Francisco, CA, USA; ^5^ California Pacific Medical Center Research Institute, San Francisco, CA, USA; ^6^ California Pacific Medical Center, San Francisco, CA, USA; ^7^ Perceptive Inc., Burlington, MA, USA; ^8^ Wake Forest University School of Medicine, Winston Salem, NC, USA; ^9^ Neuroscience Department, University of California, Berkeley, Berkeley, CA, USA

## Abstract

**Background:**

Short sleep duration is associated with elevated amyloid PET and other biomarkers of Alzheimer's disease in cognitively unimpaired older adults. Social and structural factors contribute to disparities in sleep across the lifespan, but the impact of sleep health disparities on Alzheimer's disease pathophysiology is not understood. We investigated associations between sleep duration, amyloid PET, and demographic factors in two cohorts of cognitively unimpaired older adults: US POINTER, which emphasized recruitment of individuals with sedentary lifestyles and those from under‐represented groups (URG), and A4, which did not recruit with these emphases.

**Method:**

Self‐reported sleep duration, amyloid PET imaging, and demographics were compared for US POINTER participants enrolled in both the neuroimaging and sleep (POINTERzzz) ancillary studies (*N* = 426) and A4 participants (*N* = 4485). Participants reporting sleep duration <7 hours were categorized as “short sleepers.” Amyloid positivity and centiloids were calculated using 18F‐florbetaben in US POINTER and 18F‐florbetapir in A4 with cohort‐specific processing pipelines.

**Result:**

Participants from US POINTER were younger, had fewer years of education, higher CDR sum‐of‐boxes scores, higher BMI, and were more likely to self‐identify as URG compared to A4 participants (Table). US POINTER participants had lower centiloids but the rate of amyloid positivity did not differ between studies. US POINTER participants were much more likely to be short sleepers (76%, versus 27% in A4). Shorter sleep duration was linearly associated with higher centiloids in both cohorts (Figure 1). Logistic regressions revealed that only URG status was significantly associated with short sleep (versus normal sleep) in US POINTER, whereas in A4 short sleep was associated with URG status, lower education, and marginally with higher centiloids. (Figure 2). Shorter sleep duration was significant in predicting amyloid positivity in both cohorts, as was older age. Identifying as Non‐Hispanic White was a significant predictor of amyloid positivity in A4 but was marginal in US POINTER.

**Conclusion:**

Self‐reported short sleep duration was associated with amyloid PET in two cognitively unimpaired cohorts, but with different demographic factors associated with short sleep across cohorts. Future work should determine the role of social and structural factors in sleep health, and the potential downstream effect on Alzheimer's pathophysiology.